# Predictive and Prognostic Value of Inflammatory and Nutritional Indexes in Patients with Breast Cancer Receiving Neoadjuvant Chemotherapy

**DOI:** 10.3390/medicina60111849

**Published:** 2024-11-10

**Authors:** Mustafa Ozgur Arici, Derya Kivrak Salim, Murat Kocer, Ahmet Sukru Alparslan, Baris Rafet Karakas, Banu Ozturk

**Affiliations:** 1 Department of Medical Oncology, Antalya Training and Research Hospital, 07100 Antalya, Turkey; deryakivrak@gmail.com (D.K.S.); muratkocer71@hotmail.com (M.K.); drbanutr@yahoo.com (B.O.); 2 Department of Radiology, Antalya Training and Research Hospital, 07100 Antalya, Turkey; ahmet_alparslan@yahoo.com; 3 Department of General Surgery, Antalya Training and Research Hospital, 07100 Antalya, Turkey; brkarakas@hotmail.com

**Keywords:** breast cancer, neoadjuvant chemotherapy, pan-immune inflammation value, lactate dehydrogenase-to-albumin ratio, pathologic complete response

## Abstract

*Background and Objectives*: Neoadjuvant chemotherapy (NAC) improves survival by increasing pathologic complete response (pCR). Blood-based indexes have been studied in breast cancer for predicting pCR and prognosis, but the results are conflicting. We aimed to assess the impact of inflammatory and nutritional indexes on pCR and survival. *Materials and Methods*: We retrospectively analyzed 304 patients. Pre-NAC laboratory data were used to calculate their neutrophil-to-lymphocyte ratios (NLR), pan-immune inflammation values (PIV), lactate dehydrogenase–albumin ratios (LAR), and prognostic nutritional indexes. The optimal cut-off values were determined through an analysis of the receiver operating characteristic curve. Survival analyses were performed using the Kaplan–Meier method. Multivariate regression analyses were performed to reveal the factors predicting pCR. Univariate and multivariate survival analyses were conducted to identify prognostic factors predicting survival. *Results*: The median follow-up was 38.5 months. pCR was achieved in 41.4% of the patients. In the univariate analyses, the NLR (*p* = 0.032) and PIV (*p* = 0.002) were indexes associated with pCR. In the multivariate analysis, the PIV (*p* = 0.008) was the only index significantly correlated with pCR. According to the multivariate Cox regression analyses, clinical stage 3 (*p* = 0.032), a pathologic response other than pCR (*p* = 0.021), and a high LAR (≥4.72) (*p* = 0.002) were correlated with increased recurrence risk. The univariate Cox regression analyses revealed that failure to achieve pCR (*p* = 0.037) and the presence of a high LAR (*p* = 0.044) were significant predictors of overall survival. However, the multivariate analyses failed to identify any significant predictors of death. *Conclusions*: We found that the PIV was more effective than the other indexes in predicting pCR. To our knowledge, this study is the first to determine an association between the LAR and disease-free survival in patients with breast cancer receiving NAC. We concluded that a high LAR was a poor prognostic factor, especially in patients without a pCR. Therefore, close postoperative monitoring and the intensification of adjuvant treatment should be considered for these patients. However, further studies are needed to confirm our findings.

## 1. Introduction

Breast cancer is the most frequently diagnosed malignancy and the leading cause of cancer-related mortality among women [1]. Recurrence and metastasis remain significant challenges despite recent advances in early detection and novel therapeutic modalities [2]. Neoadjuvant chemotherapy (NAC) is defined as the administration of systemic treatment prior to surgery. Achieving a pathologic complete response (pCR) after NAC is recognized as a significant prognostic marker for survival [3,4]. Historically, NAC has been considered for locally advanced or inoperable breast cancer to downstage the tumor to allow for breast-conserving surgery and potentially avoid axillary dissection [5]. NAC is increasingly being administered for operable early-stage breast cancer. However, breast cancer is a heterogeneous disease, exhibiting a range of morphologic and biological features, leading to diverse clinical behaviors and treatment responses [6]. Numerous studies have investigated the impact of various clinical and pathological factors in the response to NAC, mostly revealing differences among intrinsic subtypes in achieving pCR [7,8]. Nevertheless, more effective predictors of pCR are required to personalize treatment, optimize treatment efficacy, and minimize unnecessary toxicity.

The impact of systemic inflammation and the tumor microenvironment (TME) on treatment efficacy and prognosis in cancer has been a topic of interest in recent years. Inflammatory blood markers have been shown to predict survival and the histologic response after NAC in several tumor types, including breast cancer [9]. Inflammation indexes derived from the blood cell count, including the neutrophil-to-lymphocyte ratio (NLR), the platelet-to-lymphocyte ratio (PLR), the monocyte-to-lymphocyte ratio (MLR), the systemic immune inflammation index (SII), and the pan-immune inflammation value (PIV), have been evaluated individually and in combination [9]. Among the indexes mentioned, the PIV is notable as a comprehensive index that integrates peripheral blood immune cells such as neutrophils, lymphocytes, monocytes, and platelets. Fuca et al. first described the PIV as a prognostic biomarker for metastatic colorectal cancer in 2020 [10]. It has since become widely used for prognostic assessments in various cancers due to its simplicity and accessibility. A meta-analysis showed that a high pre-treatment PIV is associated with a poor prognosis in cancer patients [11]. The PIV has also been linked with survival and the efficacy of NAC in breast cancer [12]. It has been shown to outperform other inflammatory indexes in predicting the response to NAC and prognosis in breast cancer [13].

Nutritional immune status has also been associated with treatment response and survival. An enzyme in the glycolytic pathway, lactate dehydrogenase (LDH), has been shown to play a role in carcinogenesis by affecting the immune microenvironment [14]. Elevated pre-treatment LDH levels are associated with worse survival in cancer [15]. Albumin is a biomarker found in the peripheral blood, indicating both systemic inflammation and nutritional status [16]. The LDH–albumin ratio (LAR), along with the albumin- and lymphocyte-based prognostic nutritional index (PNI), has been researched in various cancers and acknowledged as a significant prognostic indicator [17,18,19]. However, the role of the LAR in breast cancer, particularly in patients receiving NAC, are unclear. Several studies have found a low PNI to be a poor prognostic factor in breast cancer [20,21,22]. There have been only a few studies that have evaluated the association between the PNI and pCR, with conflicting results [21,23].

The results of widely studied inflammatory and nutritional indexes are controversial. In addition, only a few studies have evaluated these simultaneously in patients with breast cancer receiving NAC [24,25]. The aim of this study was to evaluate the predictive and prognostic significance of the NLR, PIV, PNI, and LAR and their association with NAC outcomes.

## 2. Materials and Methods

### 2.1. Study Design

This study was a retrospective observational study including patients with early or locally advanced breast cancer who received NAC in our department between January 2015 and June 2023. Approval from the local ethics committee was obtained before this study was conducted.

### 2.2. Inclusion and Exclusion Criteria

A total of 304 female patients with breast cancer were included according to the following inclusion criteria:

(a) Invasive breast cancer, confirmed using core needle biopsy prior to NAC.
(b) Surgery received after NAC.
(c) Complete clinical records and follow-up information.
(d) Blood samples obtained prior to NAC.

The exclusion criteria were as follows:

(a) Patients with an inflammatory or infectious disease.
(b) Patients with missing follow-up information.
(c) Patients with bilateral breast cancer or secondary malignancy.
(d) The absence of laboratory results before NAC.

### 2.3. Data Collection, Blood Samples, and Indexes

Demographics and pre-NAC tumor characteristics, including tumor size, nodal status, clinical staging, histology, hormone receptor status, human epidermal growth factor receptor 2 (HER2) status, the Ki-67 index, and postoperative pathologic results, were recorded. The clinical staging of the patients was defined in accordance with the eighth edition of the *American Joint Committee Cancer Staging Manual* [26]. The laboratory data needed to calculate the indexes were extracted from the hospital’s electronic clinical database. For a complete blood count and biochemical analysis, two separate tubes of fresh venous blood samples were collected from the patients within two weeks prior to the administration of NAC. The samples were processed using standard automated laboratory methods, ensuring no more than a two-hour delay from the time of blood collection. Pre-NAC NLR, PIV, PNI, and LAR values were calculated based on previously published data, as follows [13,19,27,28]:

- NLR: neutrophil count (10^3^/mm^3^)/lymphocyte count (10^3^/mm^3^).
- PIV: neutrophil count (10^3^/mm^3^) × platelet count (10^3^/mm^3^) × monocyte count (10^3^/mm^3^)/lymphocyte count (10^3^/mm^3^).
- PNI: serum albumin (g/L)  +  5 × lymphocyte count (10^3^/mm^3^).
- LAR: lactate dehydrogenase (U/L)/serum albumin (g/L).

### 2.4. Treatment and Follow-Up

All patients received four to eight cycles of NAC according to international guidelines. The majority of patients received a regimen of anthracycline (A) plus cyclophosphamide (C), followed by a taxane (T) such as docetaxel or paclitaxel. In the case of HER2-positive disease, trastuzumab (H) and/or pertuzumab (P) were added to the taxane regimen (AC followed by TH or THP). A subset of HER2-positive patients was given an anthracycline-free regimen with the addition of carboplatin (Cb) as TCbH or TCbHP. After NAC, patients underwent either a mastectomy or breast-conserving surgery as clinically indicated, accompanied by either axillary lymph node dissection or sentinel lymph node biopsy. Radiation therapy was administered as indicated, and hormone-receptor-positive patients received adjuvant hormone therapy. Triple-negative and HER2-positive breast cancer subtypes that did not achieve pCR were given adjuvant capecitabine and trastuzumab emtansine, respectively. Disease-free survival (DFS) was defined as the time from the date of surgery to loco-regional recurrence, distant metastasis, or death (whichever occurred first). Overall survival (OS) was calculated from the date of diagnosis to the date of death from any cause or the last follow-up date.

### 2.5. Pathological Assessments

Patients with an estrogen receptor (ER) and/or progesterone receptor level of 1% or higher in biopsy results prior to NAC were defined as hormone-receptor-positive. HER2-positive status was identified as a CerbB2 score of +3 or +2 with a positive fluorescence in situ hybridization result. A threshold of 20% for Ki-67 was used to differentiate luminal tumors. pCR was defined as the absence of invasive cancer in both the breast and axillary lymph nodes (ypT0/ypTis, ypN0) following NAC.

### 2.6. Statistical Analysis

All statistical analyses were conducted via SPSS for Windows, version 22.0 (IBM Corp., Armonk, NY, USA). Descriptive statistics were given as numbers (n) and percentages (%) for categorical variables and median (min–max) values for continuous variables. The optimal cut-off values for the NLR, PIV, PNI, and LAR were determined using receiver operating characteristic (ROC) curve analysis for the prediction of pCR. The relationship between pCR status and clinicopathologic characteristics was evaluated using the chi-square test. A binary logistic regression analysis was conducted to determine associations between multiple variables and pCR probability. Survival analysis was performed using the Kaplan–Meier method, and the log-rank test was used to compare the variables. The multivariate Cox regression results were provided for the impact of different clinical variables on OS and DFS. A *p*-value of less than 0.05 was considered statistically significant.

## 3. Results

### 3.1. Patient and Tumor Characteristics

The study included 304 female patients with breast cancer. The median age at diagnosis was 50 years (range: 23–78 years), and 55.3% of patients were postmenopausal. The main characteristics of the patients are summarized in Table 1.

### 3.2. ROC Analysis

Table 2 shows the optimal cut-off values for predicting pCR for each index. Patients are categorized based on values for each index, which are determined using ROC curve analysis.

### 3.3. Predictive Factors for pCR

pCR was achieved in 41.4% of the patients. The highest pCR rate was observed in hormone-receptor-negative, HER2-positive disease (77.7%), followed by luminal B HER2-positive patients (60.7%). Among 66 triple-negative patients, 35 (53.0%) had pCR. Luminal B HER2-negative and luminal A patients had the lowest pCR rates (23.6% and 10%, respectively). Table 3 shows the association of pCR status with clinicopathologic parameters. The clinical stage, pre-treatment histology, ER status, HER2 status, biological subtypes, Ki-67 group, NLR, and PIV were significantly associated with the response to NAC.

Univariate and multivariate logistic regression analyses were performed to determine the predictors of pCR (Table 4). According to univariate analyses, clinical stage (*p* = 0.018), ER status (*p =* 0.001), HER2 status (*p* = 0.001), a high Ki-67 index (*p* = 0.002), a high NLR (*p* = 0.032), and a low PIV (*p* = 0.002) were associated with a higher probability of achieving pCR. In the multivariate analyses, the PIV was the only blood-based index significantly correlated with pCR (*p* = 0.008). Clinical stage (*p* = 0.020), ER status (*p* = 0.001), HER2 status (*p* = 0.001), and a high Ki-67 index (*p* = 0.036) were also found to be other independent predictors.

### 3.4. Univariate and Multivariate Analyses for DFS and OS

At a median follow-up of 38.5 months, 34 patients (11.2%) had experienced a recurrence, and 17 patients (5.6%) had died. Median DFS and OS were not reached.

Table 5 shows the results of univariate and multivariate Cox regression analyses for the predictors of recurrence. Clinical stage 3 (*p* = 0.032), failure to achieve pCR (*p* = 0.021), and a high LAR (≥4.72) (*p* = 0.002) were correlated with a higher risk of recurrence in multivariate analysis. Regarding OS, non-pCR and a high LAR were significant predictors for OS (*p* = 0.037 and *p* = 0.044, respectively). There was no independent predictor of death in the multivariate analysis (Table 6).

### 3.5. Kaplan–Meier Survival Analyses

Achieving a pCR had a significant impact on both DFS (*p* = 0.004) and OS (*p* = 0.021). The 60-month DFS and OS rates were 93% and 97.1% for patients with pCR and 74.8% and 89.9% for non-pCR, respectively (Supplementary Materials, Figures S1 and S2).

**Figure 1:**
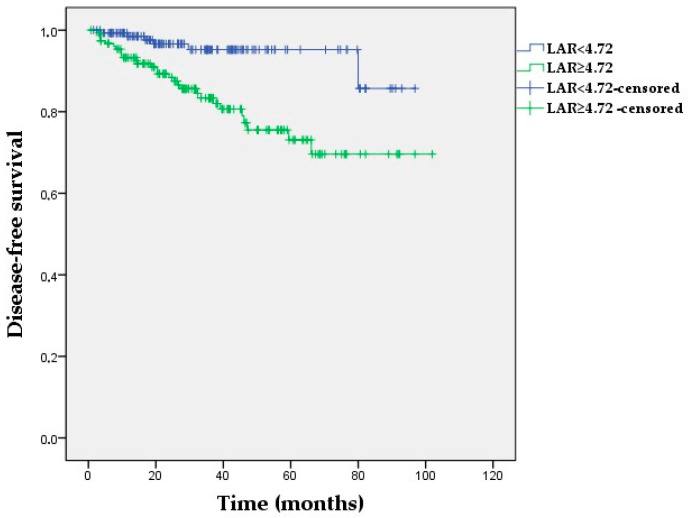
Kaplan–Meier survival curves for disease-free survival according to low vs. high LAR in the study population (log-rank *p* = 0.001).

For DFS, Kaplan–Meier survival analyses revealed no significant differences among the NLR, PIV, and PNI groups (*p* = 0.055, *p* = 0.180, and *p* = 0.099, respectively). The 60-month DFS was 95.2% and 73.1% for the low- and high-LAR groups, respectively (*p* = 0.001) (Figure 1). The DFS benefit for the low-LAR group remained significant in the non-pCR cohort when the patients were categorized using pCR status (*p* = 0.003). However, median DFS was similar between the low- and high-LAR groups among the patients who achieved pCR (*p* = 0.271) (Supplementary Materials, Figures S3 and S4).

**Figure 2:**
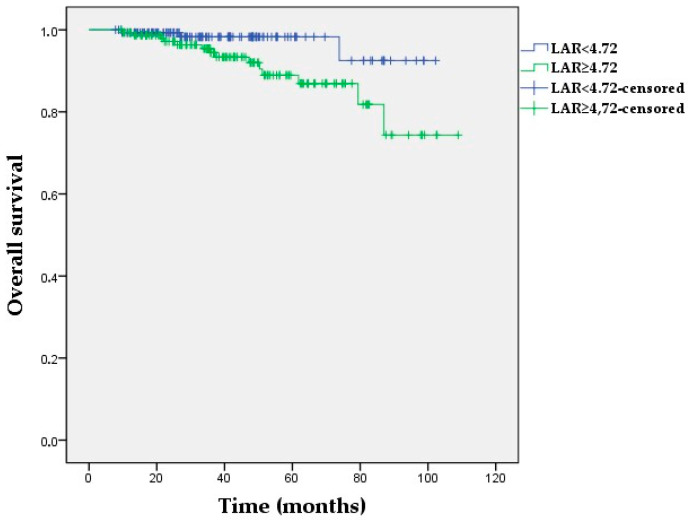
Kaplan–Meier survival curves for overall survival according to low vs. high LAR in the study population (log-rank *p* = 0.031).

**Figure 3:**
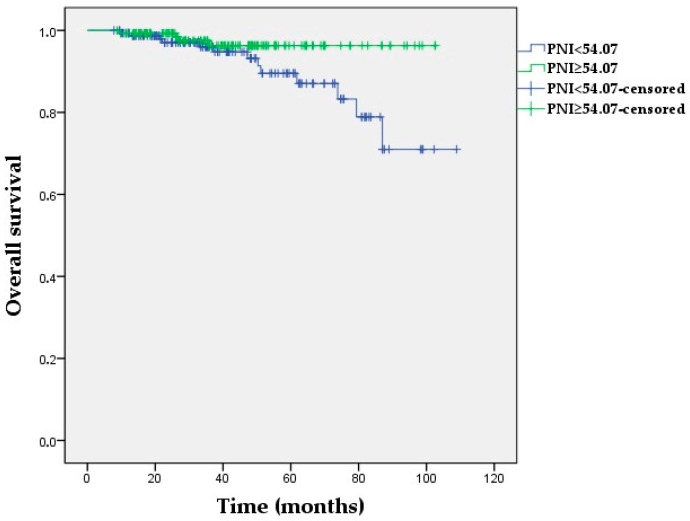
Kaplan–Meier survival curves for overall survival according to low vs. high PNI in the study population (log-rank *p* = 0.045).

The Kaplan–Meier OS analyses demonstrated no statistically significant differences in the NLR and PIV groups (*p =* 0.177, *p =* 0.450, respectively). The 60-month OS was 98% and 88.9% for the low- and high-LAR groups, respectively (*p =* 0.031) (Figure 2). Furthermore, a low LAR was significantly associated with longer OS in non-pCR patients (*p =* 0.045), whereas the OS was not affected by LAR in patients with pCR (p = 0.952) (Supplementary Materials, Figures S5 and S6). A high PNI was correlated with longer OS compared with a low PNI (*p =* 0.045) (Figure 3). Additionally, median OS in the high-PNI group was significantly prolonged in non-pCR patients (*p =* 0.028), whereas there was no association between OS and the PNI in patients who achieved pCR (*p =* 0.995) (Supplementary Materials, Figures S7 and S8).

## 4. Discussion

In this study, we investigated inflammatory and nutritional indexes concurrently in order to identify the most efficacious index for predicting pCR and survival. We found that the PIV was the only significant predictor for pCR. However, there was no association between the PIV and survival. Only pCR and the LAR were identified as independent predictors of recurrence risk.

In recent years, the roles of inflammation and the TME in the different stages of carcinogenesis and the failure of anticancer therapy have been well recognized [29]. The TME comprises a variety of immune cells, including neutrophils, monocytes, and lymphocytes. Additionally, stromal cells and platelets are other essential components of the TME [30]. Considering the role of these types of cells in cancer, various inflammatory indexes (NLR, PLR, MLR, SII, PIV, etc.) have been established. The results of studies in predicting treatment response and prognosis in breast cancer using inflammatory indexes are inconsistent, even when the same index is considered [9]. Nutritional status is another critical factor in the development and prognosis of cancer [31]. Indexes reflecting both inflammation and nutritional status, such as PNI and LAR, have already been defined. However, few studies have evaluated these inflammatory and nutritional indexes together to define the most effective index [24,25].

Combining all routinely assessed blood cell populations, including neutrophils, monocytes, lymphocytes, and platelets, the PIV reflects systemic inflammation and immunity. Sahin et al. found that PIV was a promising predictor of pCR in breast cancer and also reported that PIV showed better predictive value than NLR, PLR, MLR, and SII [13]. A recent study also confirmed a low PIV as an independent predictor for achieving axillary pCR in patients with breast cancer receiving NAC [32]. In concordance with these studies, we found that the PIV was more powerful than other blood-based indexes for predicting pCR in patients with breast cancer receiving NAC. According to a meta-analysis involving several types of cancer, patients with a high PIV had worse survival than patients with a low PIV [11]. However, the results in breast cancer were conflicting. Qi et al. suggested that a high PIV was associated with worse survival [12], whereas Truffi et al. found no correlation between the PIV and distant metastasis-free survival [33]. Furthermore, PIV was not associated with survival in another study that analyzed inflammatory and nutritional indexes together [24]. Although a high PIV was a powerful predictive factor of pCR, the PIV failed to show the same power in survival analysis in our study when analyzed together with nutritional indexes. As an index reflecting acute inflammation, the PIV may be expected to predict pCR, which is an early end point in neoadjuvant studies. However, long-term survival outcomes were not affected by the PIV in our study, suggesting that chronic inflammation and nutritional status may be more important factors.

As tumor cells grow abnormally, they consume more oxygen and depend on anaerobic glycolysis to provide energy. On the other hand, even with adequate oxygen, tumor cells preferentially metabolize glucose through glycolysis to generate energy for rapid proliferation [34]. LDH plays an important role in glycolysis by converting pyruvate to lactate. It was found that the serum LDH level rises both as an indicator of increased glycolysis and as a result of the hypoxic process [15,35]. Moreover, Ding et al. discovered that LDH enables tumor cells to suppress and evade the immune system by altering the TME [14]. In a meta-analysis consisting mainly of metastatic breast cancer studies, high serum LDH levels were poor prognostic factors [36]. Albumin is a powerful index of the systemic nutritional status in cancer patients. Alterations in serum albumin levels may indicate the disease severity, progression, and prognosis [37,38]. These findings suggest that the LAR could be a powerful index for the thorough assessment of nutritional status, systemic inflammation, and tumor burden. The LAR has been shown to be an independent prognostic indicator in several cancers [39,40,41,42]. However, the prognostic value of the LAR in breast cancer has not been well established. In a study of 134 patients with breast cancer who underwent upfront surgery, a high preoperative LAR was associated with poor DFS, whereas the PLR and MLR inflammatory indexes were not [19]. To the best of our knowledge, our study is the first to demonstrate an association between the LAR and DFS in patients with breast cancer undergoing NAC.

The current study demonstrated that a high LAR is an independent prognostic factor for DFS and OS. Based on Kaplan–Meier survival analysis, a high LAR was significantly associated with worse DFS in patients who failed to achieve pCR. Conversely, no significant difference in DFS was observed in patients who achieved pCR. One possible explanation for this is that pCR, regardless of the LAR, is the most significant predictor of a favorable prognosis. However, in the absence of pCR, the strength of the LAR turns out to be a significant factor. We concluded that these patients (i.e., non-pCR and a high LAR) should be treated more intensively after surgery and that closer monitoring for relapse is critical.

The PNI, which is derived from albumin and lymphocyte counts, is another index that indicates both nutritional and immune status [17]. Initially used to assess the nutritional and immunogenic status of patients prior to surgery, the PNI has potential value in providing prognostic information for many cancers [17]. Several studies have investigated the prognostic value of PNI in breast cancer and found that a low PNI was a poor prognostic factor [20,21,22]. In two recent meta-analyses, the PNI was found to be associated with OS but not with DFS in breast cancer [43,44]. There have been few studies about the PNI and treatment outcomes in patients with breast cancer receiving NAC. Chen et al. demonstrated that a high PNI was an independent predictive factor for both better DFS and OS in patients with breast cancer undergoing NAC [28]. Another study assessing inflammatory and nutritional indexes concurrently found results that were similar to those published by Chen et al. [24]. Our study found that a high PNI was associated with increased survival. However, PNI had lost its significance after multivariate analysis.

On the other hand, studies investigating the relationship between PNI and pCR have shown conflicting results. One study found that a high PNI (≥53) was associated with a significantly increased pCR rate [23]. Wang et al. reported contrary results and stated that patients with an extremely high PNI (>55) had more difficulty achieving pCR [21]. In our analysis, the PNI and LAR demonstrated inferior predictive ability in predicting pCR when compared to PIV. This result may be explained using the previously mentioned hypothesis that pCR is an early end point that is best represented by indexes of inflammation rather than nutrition.

Our study has some limitations. First, this is a retrospective, single-center study. Second, selection bias cannot be avoided even if eligibility criteria are implemented to minimize bias. Third, due to the extensive study period and the subsequent evolution of NAC protocols over time, it is possible that the heterogeneity of NAC regimens may exist among patients, reducing the generalizability of the results. To minimize potential bias, further studies with proper patient grouping based on treatment should be conducted to analyze indexes. Despite these limitations, our pCR rate was consistent with the literature and meta-analyses. In addition, the similarity of treatment regimens eliminated the confounding effect of treatment.

## 5. Conclusions

Our study demonstrated that the PIV was more effective than other indexes in predicting pCR. Furthermore, to our knowledge, this study is the first to demonstrate an association between the LAR and DFS in patients with breast cancer receiving NAC. We concluded that the PIV and LAR can be used as predictors of treatment outcome; in the absence of pCR, in particular, a high LAR (≥4.72) was associated with poor prognosis. Therefore, close monitoring with more aggressive adjuvant treatment options should be considered for these patients. However, further studies are needed to confirm our findings.

## Figures and Tables

**Table 1:** Main characteristics of the patients.

Variable	n (%)
Age	
Median (range), years	50 (23–78)
Menopausal status	
Premenopausal	136 (44.7)
Postmenopausal	168 (55.3)
Clinical stage	
Stage II	154 (50.7)
Stage III	150 (49.3)
Pre-treatment histology	
Invasive carcinoma, NST	274 (90.1)
Others *	30 (9.9)
ER status	
Positive	196 (64.5)
Negative	108 (35.5)
HER2 status	
Positive	92 (30.3)
Negative	212 (69.7)
Biological subtype	
Lum A	40 (13.2)
Lum B, HER2-negative	106 (34.9)
Lum B, HER2-positive	56 (18.4)
HR-negative, HER2-positive	36 (11.8)
Triple-negative	66 (21.7)
Ki-67 index (%)	
Median (range)	30 (3–100)
NAC regimen	
Anthracycline + Taxane	207 (68.1)
Anthracycline + Taxane + Anti HER2	75 (24.7)
Anthracycline-free	22 (7.2)
pCR status	
pCR	126 (41.4)
Non-pCR	178 (58.6)

NST: no special type; ER: estrogen receptor; HER2: human epidermal growth factor receptor 2; Lum: luminal; HR: hormone receptor; NAC: neoadjuvant chemotherapy; pCR: pathologic complete response. * Includes lobular, mucinous, papillary, and metaplastic tumors.

**Table 2:** Results of receiver operating characteristics curve analysis for pathologic complete response.

	Optimal Cut-Off	AUC	95% CI	Specificity (%)	Sensitivity (%)	*p-*Value
NLR	2.09	0.587	0.523–0.652	56.3	56.2	0.009
PIV	317.5	0.618	0.556–0.681	58.7	59.0	<0.001
PNI	54.07	0.455	0.389–0.520	46.8	45.5	0.177
LAR	4.72	0.579	0.514–0.645	55.6	55.6	0.018

AUC: area under the curve; CI: confidence interval; NLR: neutrophil–lymphocyte ratio; PIV: pan-immune inflammation value; PNI: prognostic nutritional index; LAR: lactate dehydrogenase–albumin ratio.

**Table 3:** Association of patient characteristics with pathologic response.

	pCR (n = 126)	Non-pCR (n = 178)	*p-*Value
Age group			0.343
<50	65 (51.6)	82 (46.1)	
≥50	61 (48.4)	96 (53.9)	
Menopausal status			0.187
Premenopausal	62 (49.2)	74 (41.6)	
Postmenopausal	64 (50.8)	104 (58.4)	
Clinical stage			0.018
Stage II	74 (58.7)	80 (44.9)	
Stage III	52 (41.3)	98 (55.1)	
Pre-treatment histology			0.004
Invasive carcinoma, NST	121 (96.0)	153 (86.0)	
Others	5 (4.0)	25 (14.0)	
ER status			<0.001
Positive	58 (46.0)	138 (77.5)	
Negative	68 (54.0)	40 (22.5)	
HER2 status			<0.001
Positive	62 (49.2)	30 (16.9)	
Negative	64 (50.8)	148 (83.1)	
Biological subtypes			<0.001
Lum A	4 (3.2)	36 (20.2)	
Lum B, HER-2-negative	25 (19.8)	81 (45.5)	
Lum B, HER-2-positive	34 (27.0)	22 (12.4)	
HR-negative, HER2-positive	28 (22.2)	8 (4.5)	
Triple-negative	35 (27.8)	31 (17.4)	
Ki-67 group			0.001
<20%	13 (10.3)	45 (25.3)	
≥20%	113 (89.7)	133 (74.7)	
NLR			0.031
<2.09	71 (56.3)	78 (43.8)	
≥2.09	55 (43.7)	100 (56.2)	
PIV			0.002
<317.5	74 (58.7)	73 (41.0)	
≥317.5	52 (41.3)	105 (59.0)	
PNI			0.188
<54.07	59 (46.8)	97 (54.5)	
≥54.07	67 (53.2)	81 (45.5)	
LAR			0.055
<4.72	70 (55.6)	79 (44.4)	
≥4.72	56 (44.4)	99 (55.6)	

pCR: pathologic complete response; NST: no special type; ER: estrogen receptor; HER2: human epidermal growth factor receptor 2; Lum: luminal; HR: hormone receptor; NLR: neutrophil–lymphocyte ratio; PIV: pan-immune inflammation value; PNI: prognostic nutritional index; LAR: lactate dehydrogenase–albumin ratio. Pearson’s chi-squared test. Numbers in bold indicate statistical significance.

**Table 4:** Logistic regression analyses for pCR.

	Univariate		Multivariate	
Risk Factor	OR (95%CI)	*p-*Value	OR (95%CI)	*p-*Value
Age group (>50 vs. ≤50)	0.80 (0.5–1.26)	0.343		
Clinical stage (Stage 3 vs. 2)	0.57 (0.36–0.91)	**0.018**	0.52 (0.31–0.90)	**0.020**
ER (negative vs. positive)	4.05 (2.46–6.46)	**0.001**	3.77 (2.17–6.54)	**0.001**
HER2 (negative vs. positive)	4.78 (2.82–8.08)	**0.001**	5.56 (3.10–9.96)	**0.001**
Ki-67 (≥20 vs. <20)	2.95 (1.51–5.72)	**0.002**	2.26 (1.05–4.85)	**0.036**
NLR (≥2.09 vs. <2.09)	0.60 (0.38–0.95)	**0.032**	0.89 (0.50–1.59)	0.697
PIV (<317.5 vs. ≥317.5)	2.04 (1.28–3.25)	**0.002**	2.06 (1.20–3.52)	**0.008**
PNI (≥54.07 vs. <54.07)	0.19 (0.86–2.15)	0.188		
LAR (≥4.72 vs. <4.72)	0.64 (0.40–1.01)	0.055		

ER: estrogen receptor; HER2: human epidermal growth factor receptor 2; NLR: neutrophil-to-lymphocyte ratio; PIV: pan-immune inflammation value; PNI: prognostic nutritional index; LAR: lactate dehydrogenase-to-albumin ratio; OR: odds ratio; CI: confidence interval. Numbers in bold indicate statistical significance.

**Table 5:** Cox regression analysis for the predictors of recurrence.

	Univariate		Multivariate	
Risk Factor	OR (95%CI)	*p-*Value	OR (95%CI)	*p-*Value
Age group (>50 vs. ≤50)	0.88 (0.44–1.72)	0.713		
Clinical stage (Stage 3 vs. 2)	2.18 (1.06–4.48)	**0.033**	2.20 (1.07–4.54)	**0.032**
ER (negative vs. positive)	1.55 (0.78–3.08)	0.208		
HER2 (negative vs. positive)	1.37 (0.67–2.78)	0.378		
Ki-67 (≥20 vs. <20)	2.18 (0.83–5.69)	0.111		
Pathologic status (non-pCR vs. pCR)	3.40 (1.40–8.22)	**0.006**	2.84 (1.17–6.88)	**0.021**
NLR (≥2.09 vs. <2.09)	2.20 (0.96–4.24)	0.061		
PIV (≥317.5 vs. <317.5)	1.61 (0.79–3.25)	0.184		
PNI (≥54.07 vs. <54.07)	0.55 (0.27–1.12)	0.104		
LAR (≥4.72 vs. <4.72)	4.08 (1.68–9.86)	**0.002**	3.98 (1.64–9.65)	**0.002**

ER: estrogen receptor; HER2: human epidermal growth factor receptor 2; NLR: neutrophil-to-lymphocyte ratio; PIV: pan-immune inflammation value; PNI: prognostic nutritional index; LAR: lactate dehydrogenase-to-albumin ratio; OR: odds ratio; CI: confidence interval. Numbers in bold indicate statistical significance.

**Table 6:** Cox regression analysis for the predictors of death.

	Univariate		Multivariate	
Risk Factor	OR (95%CI)	*p-*Value	OR (95%CI)	*p-*Value
Age group (>50 vs. ≤50)	1.12 (0.43–2.91)	0.811		
Clinical stage (Stage 3 vs. 2)	1.70 (0.62–4.62)	0.294		
ER (negative vs. positive)	0.85 (0.31–2.31)	0.755		
HER2 (negative vs. positive)	0.74 (0.24–2.29)	0.608		
Ki-67 (≥20 vs. <20)	3.12 (0.70–13.79)	0.134		
Pathologic status (non-pCR vs. pCR)	4.83 (1.10–21.16)	**0.037**	4.29 (0.98–18.86)	0.054
NLR (≥2.09 vs. <2.09)	2.39 (0.77–7.38)	0.104		
PIV (≥317.5 vs. <317.5)	1.46 (0.54–3.97)	0.453		
PNI (<54.07 vs. ≥54.07)	2.99 (0.97–9.18)	0.056		
LAR (≥4.72 vs. <4.72)	3.61(1.03–12.61)	**0.044**	3.17 (0.90–11.10)	0.071

ER: estrogen receptor; HER2: human epidermal growth factor receptor 2; NLR: neutrophil–lymphocyte ratio; PIV: pan-immune inflammation value; PNI: prognostic nutritional index; LAR: lactate dehydrogenase–albumin ratio; OR: odds ratio; CI: confidence interval. Numbers in bold indicate statistical significance.

## Data Availability

The data presented in this study are available upon request from the corresponding author due to privacy and/or ethical restrictions.

## Supplementary Materials

The following supporting information can be downloaded at https://www.mdpi.com/article/10.3390/medicina60111849/s1: Figure S1: Disease-free survival according to pathologic complete response (pCR) status; Figure S2: Overall survival according to pathologic complete response (pCR) status; Figure S3: Disease-free survival according to LAR status in patients with residual disease (non-pCR); Figure S4: Disease-free survival according to LAR status in patients with pathologic complete response (pCR); Figure S5: Overall survival according to LAR status in patients with residual disease (non-pCR); Figure S6: Overall survival according to LAR status in patients with pathologic complete response (pCR); Figure S7: Overall survival according to PNI status in patients with residual disease (non-pCR); Figure S8: Overall survival according to PNI status in patients with pathologic complete response (pCR).
