# Supplementary material for: Predictive and Prognostic Value of Inflammatory and Nutritional Indexes in Patients with Breast Cancer Receiving Neoadjuvant Chemotherapy
Source: Medicina (Kaunas). 2024 Nov 10;60(11):1849. doi: 10.3390/medicina60111849 (PMC11596226; doi:10.3390/medicina60111849)
Supplement: Supplementary file 1 [file medicina-60-01849-s001.zip › medicina-3288250-supplementary.pdf]

Supplementary Figures:

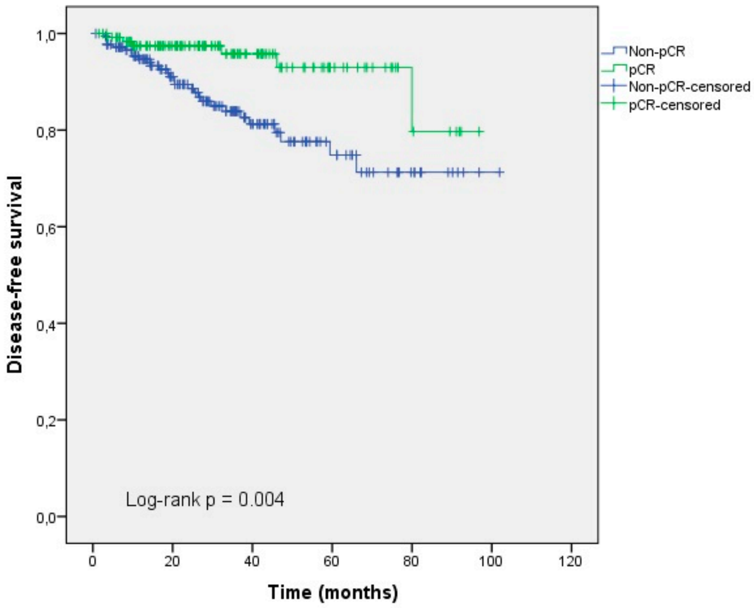

Supplementary Figure S1. Disease-free survival according to pathologic complete response (pCR) status

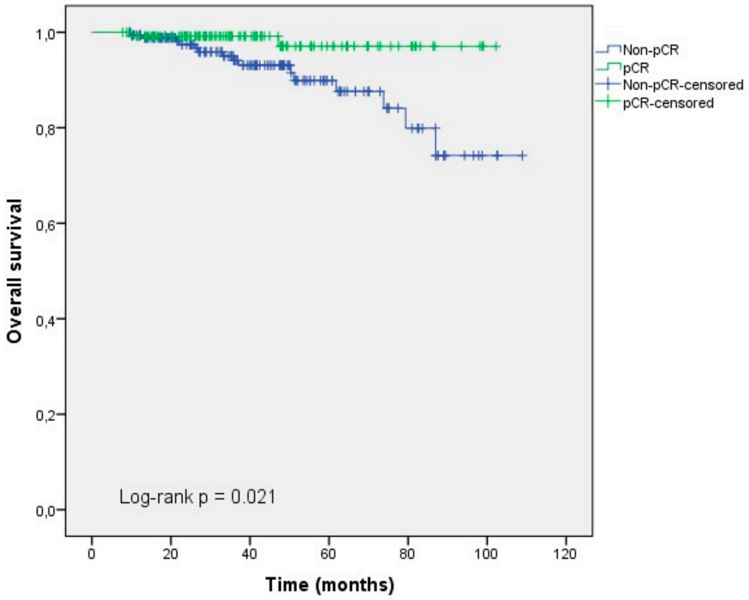

Supplementary Figure S2. Overall survival according to pathologic complete response (pCR) status

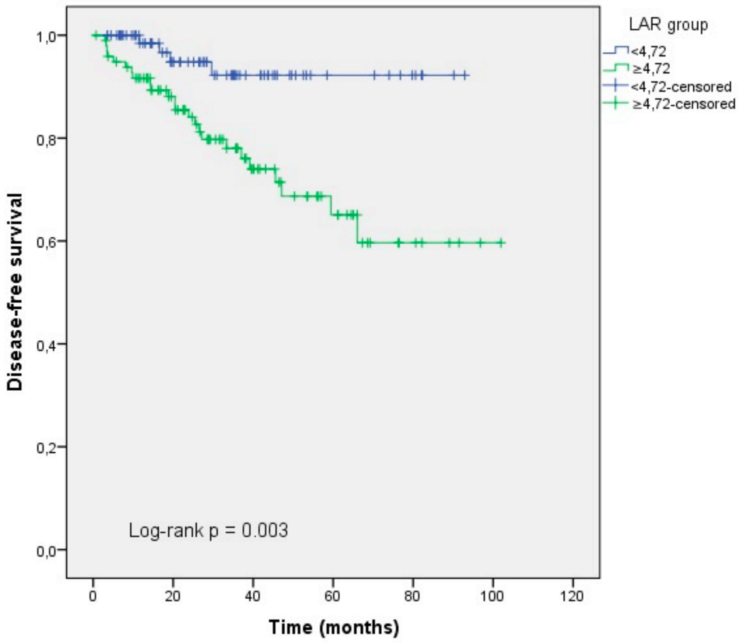

Supplementary Figure S3. Disease-free survival according to LAR status in patients with residual disease (non-pCR)

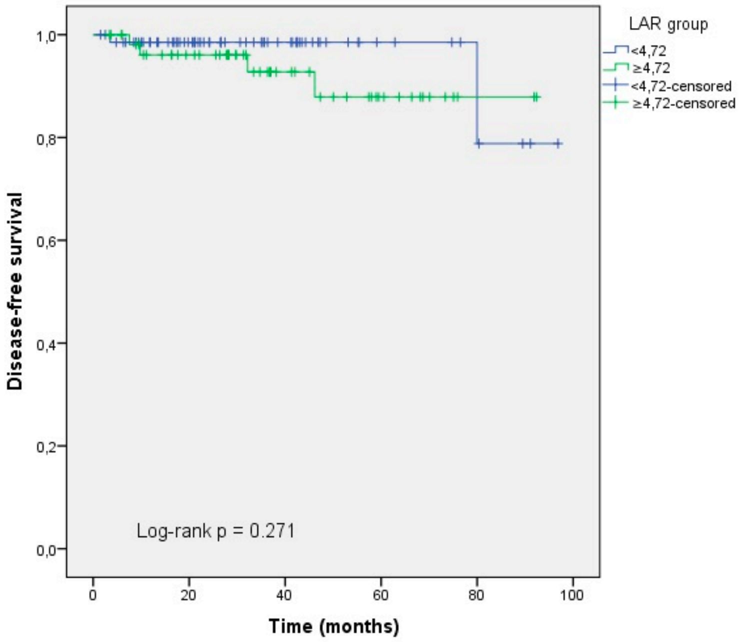

Supplementary Figure S4. Disease-free survival according to LAR status in patients with pathologic complete response (pCR)

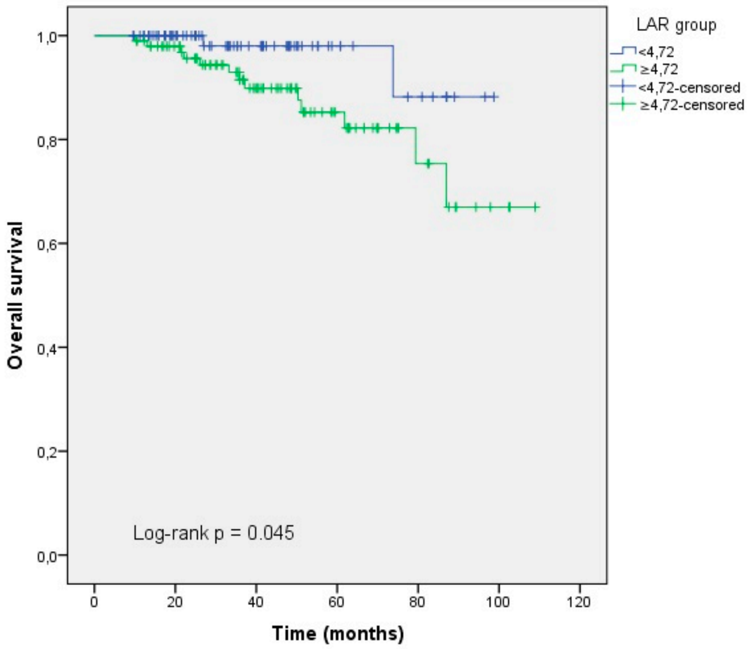

Supplementary Figure S5. Overall survival according to LAR status in patients with residual disease (non-pCR)

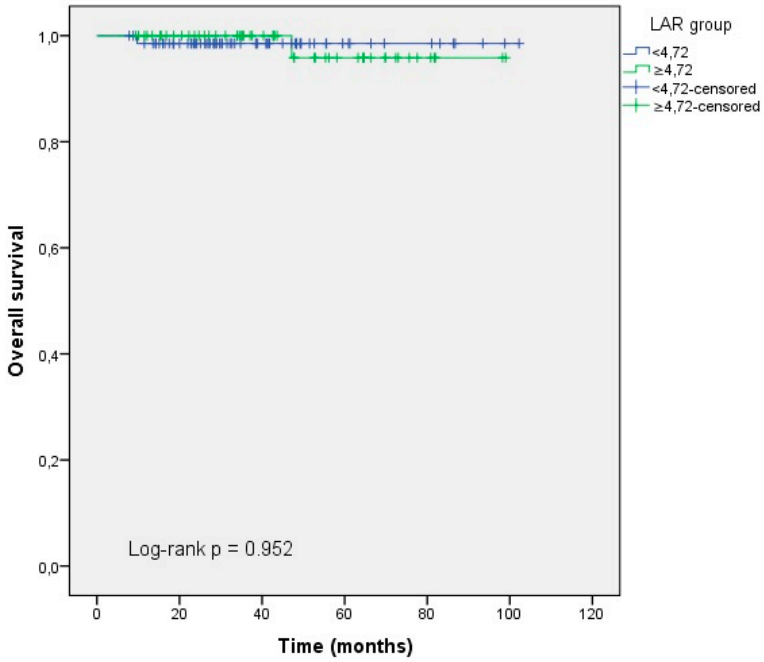

Supplementary Figure S6. Overall survival according to LAR status in patients with pathologic complete response (pCR)

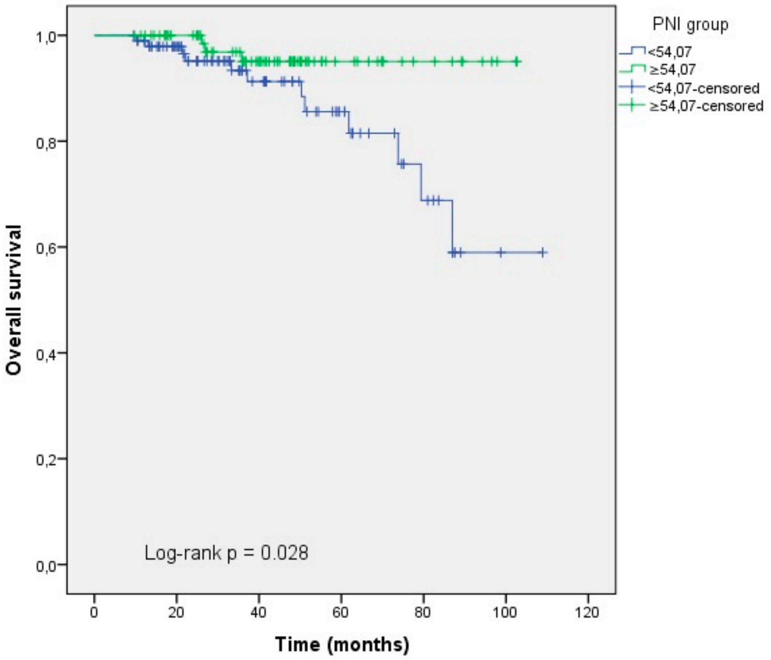

Supplementary Figure S7. Overall survival according to PNI status in patients with residual disease (non-pCR)

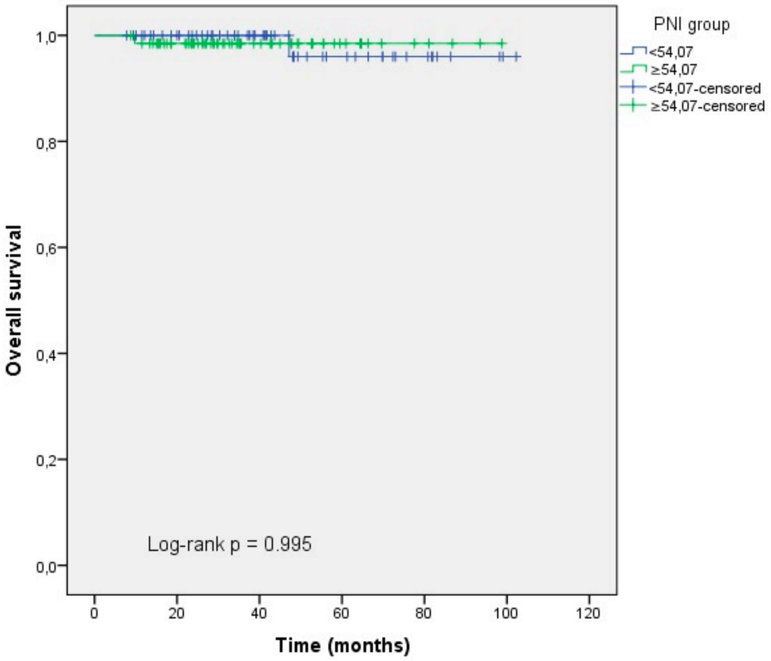

Supplementary Figure S8. Overall survival according to PNI status in patients with pathologic complete response (pCR)
